# Allicin Inhibits Porcine Reproductive and Respiratory Syndrome Virus Infection In Vitro and Alleviates Inflammatory Responses

**DOI:** 10.3390/v15051050

**Published:** 2023-04-25

**Authors:** Jingbo Hu, Chenxi Li, Yanyang Zhou, Jingjing Ding, Xiangdong Li, Yanhua Li

**Affiliations:** 1 College of Veterinary Medicine, Yangzhou University, Yangzhou 225009, China; 2 Comparative Medicine Research Institute, Yangzhou University, Yangzhou 225009, China; 3 Jiangsu Co-Innovation Center for Prevention and Control of Important Animal Infectious Diseases and Zoonoses, Yangzhou 225009, China

**Keywords:** porcine reproductive and respiratory syndrome virus, allicin, viral entry, viral replication, anti-inflammatory

## Abstract

Porcine reproductive and respiratory syndrome virus (PRRSV) is one of the most economically important pathogens to the swine industry worldwide over the past three decades. No approved effective antiviral drug is available to control this virus. The antiviral effects of allicin (diallyl thiosulfinate) on many human and animal viruses have been documented. However, the antiviral effect of allicin on PRRSV infection remains unknown. In this study, we found that allicin exhibited an inhibitory effect on HP-PRRSV and NADC30-like PRRSV in a dose-dependent manner by interfering with viral entry, replication, and assembly. Furthermore, allicin alleviated the expression of pro-inflammatory cytokines (IFN-β, IL-6, and TNFα) induced by PRRSV infection. The pro-inflammatory signaling pathways, TNF signaling pathway and MAPK signaling pathway, up-regulated by PRRSV infection were restored by allicin treatment. Taken together, these results demonstrate that allicin has antiviral activity against PRRSV and ameliorates inflammatory responses induced by PRRSV infection, suggesting that allicin is a promising drug candidate for anti-PRRSV therapy in vivo.

## 1. Introduction

Porcine reproductive and respiratory syndrome (PRRS) is one of the most economically important infectious diseases to the swine industry worldwide. This disease is characterized by the serious reproductive failure of pregnant sows and respiratory symptoms of growing pigs. In diseased animals, interstitial pneumonia caused by excessive production of pro-inflammatory cytokines in the lungs is always associated with high mortality [1,2]. The etiological agent of PRRS is the porcine reproductive and respiratory syndrome virus (PRRSV), an enveloped single-stranded positive-sense RNA virus in the Arteriviridae family, Nidovirales order. According to the latest virus taxonomy (https://ictv.global/ (accessed on 29 March 2023)), PRRSV has two species, PRRSV-1 (*Betaarterivirus suid* 1) and PRRSV-2 (*Betaarterivirus suid* 2). They outbroke almost simultaneously in the United States and Europe [3] and were initially recognized as two genotypes. During the last three decades, PRRSV has been widespread and has caused tremendous economic losses to the swine industry worldwide. In China, the predominant PRRSV strains in the herd belong to PRRSV-2, although the PRRSV-1 strains have been isolated sporadically. The extremely high mutation rate of the PRRSV genome [4,5] and RNA recombination between different strains [6] have led to the emergence of new PRRSV strains almost every few years, such as HP-PRRSV strains in Asian countries [7], NADC30-like strains in China [8], and NADC34-like strains in China [9]. Although vaccination is the most effective strategy for PRRS control, commercial vaccines provide limited protection against heterologous infection [10,11]. To date, no specific antiviral therapy is available for PRRS control. Therefore, it is urgent to develop antiviral drugs to block infection or alleviate inflammatory responses induced by PRRSV infection.

Garlic and its organosulfur compounds (OSC) have many pharmacological properties, such as antibacterial, antiviral, anti-inflammatory, anticancer, and antioxidant properties [12,13,14,15]. Allicin (diallyl thiosulfinate) is an OSC found in garlic, onion, and other Allium plants [16,17]. Weber et al. reported that allicin and its derivatives contribute significantly to the antiviral properties of allicin against herpes simplex virus-1 and 2, parainfluenza-3, vaccinia virus, vesicular stomatitis virus, and human rhinovirus-2 [18]. The antiviral effect of allicin against several respiratory viruses, including influenza, SARS-CoV, and rhinovirus, has been investigated [15]. In a recent study by Mösbauer et al., allicin alleviated the infection of SARS-CoV-2 in vitro and restored the host cellular pathways induced by viral infection [19]. Similarly, allicin exerts an antiviral effect against the reticuloendotheliosis virus by restricting inflammation and oxidative damage caused by the infection, especially by blocking the ERK/MAPK pathway [20]. Inhibition of the P38 and JNK pathways and the TLR4/NF-*κ*B signaling pathway was also considered as the anti-inflammatory mechanism of allicin [14,21].

Supplementation of garlic botanicals in the nursery basal diet reduced viral loads in PRRSV-infected pigs and improved the immune responses of pigs [22]. However, the effect of allicin on PRRSV infection has not been explored. In this study, we investigated the antiviral effect of allicin on PRRSV infection in vitro and characterized potential mechanisms. Our results indicated that allicin inhibits PRRSV infection by blocking multiple stages in the viral life cycle, including viral entry, replication, and assembly. The expression of pro-inflammatory cytokines induced by PRRSV infection was alleviated by allicin treatment. The anti-inflammatory effect of allicin in PRRSV-infected cells was confirmed by transcriptome analysis. The enhanced TNF signaling pathway and MAPK signaling pathways in PRRSV-infected cells were restored by allicin treatment. Therefore, allicin could be a promising drug candidate for PRRS control due to its antiviral and anti-inflammatory effects.

## 2. Materials and Methods

### 2.1. Cells, Viruses, Antibodies, and Compounds

MARC-145 cells (ATCC) were cultured in Modified Eagle Medium (MEM; Sigma-Aldrich, St. Louis, MO, USA) containing 10% fetal bovine serum (FBS; Sigma-Aldrich, St. Louis, MO, USA) and 1% penicillin-streptomycin (Thermo Fisher Scientific, Waltham, MA, USA). BHK-21 cells (ATCC) were maintained in Dulbecco’s modified eagle medium (DMEM; Sigma-Aldrich, St. Louis, MO, USA) supplemented with 10% FBS (Gemini Bio, West Sacramento, CA, USA) and 1% penicillin-streptomycin. Porcine alveolar macrophages (PAM) were isolated from PRRSV-negative piglets bronchoalveolar lavage fluid, and cultured in RPMI-1640 medium (Sigma-Aldrich, St. Louis, MO, USA) containing 10% FBS (Sigma-Aldrich, St. Louis, MO, USA) and 2% penicillin-streptomycin. All cells were cultured at 37 °C with 5% CO_2_ in a humidified incubator (ThermoFisher Scientific, Waltham, MA, USA).

Three PRRSV strains were used in this study, including NADC30-like PRRSV NL1207 strain (GenBank accession No. MZ399800.1), HP-PRRSV BLC-2 strain, and HP-PRRSV TA-12 strain (GenBank Accession No. HQ416720.1). Two reporter viruses (TA-EGFP and TA-Gluc2) derived from TA-12 were also used [8]. A monoclonal antibody against PRRSV N protein was purchased from MEDIAN Diagnostics, Korea. A monoclonal antibody against β-actin was purchased from HuaBio (Hangzhou, China). Rabbit monoclonal antibodies against Rab5 and Rab7 were purchased from Beyotime (Shanghai, China). A rabbit polyclonal antibody against PRRSV nsp2 was generously provided by Dr. Zuzhang Wei from Guangxi University. A monoclonal antibody against porcine CD163 was kindly provided by Dr. Fei Gao from Shanghai Veterinary Research Institute. Allicin with purity > 99% from MedChemExpress (Shanghai, China) was dissolved in dimethylsulfoxide (DMSO) to 100 mg/mL stock and stored at −80 °C.

### 2.2. Cytotoxicity Assay

The cytotoxic effect of allicin on MARC-145 cells, PAMs, or BHK-21 cells was evaluated using the Cell Counting Kit-8 (Vazyme Biotech, Nanjing, China). Cell monolayers in 96-well plates were treated with allicin at 0, 10, 20, 40, 80, 100, 200, and 400 μg/mL for 24 h at 37 °C. Cell monolayers were washed with PBS three times and incubated with MEM supplemented with 10% CCK8 for 1 h at 37 °C. Subsequently, cell viability was determined by absorbance at 450 nm measured with a microplate reader (Gallop Technology, Shanghai, China).

### 2.3. Quantitative Real-Time PCR (qRT-PCR)

We performed qRT-PCR to evaluate the viral load and the level of cytokine gene expression at the mRNA level using a QuantGene 9600 real-time PCR system (Bioer Technology, Hangzhou, China). For viral load quantification, viral RNA in the virus supernatant was extracted using the TIANamp Virus RNA Kit (TIANGEN, Beijing, China) according to the manufacturer’s instructions. One-step qRT-PCR was conducted with HiScript II U+ One Step qRT-PCR Probe Kit (Vazyme Biotech, Nanjing, China) using the primers/probe set targeting PRRSV ORF6 (Table 1). For quantification of the mRNA level of viral and cellular genes, total cellular RNA was extracted from cultured cells using FastPure Cell/Tissue Total RNA Isolation Kit V2 (Vazyme Biotech, Nanjing, China). The cDNA synthesized with a Hiscript II 1st Strand cDNA Synthesis Kit (Vazyme Biotech, Nanjing, China) was used as the template for qPCR with AceQ U+ Probe Master Mix or AceQ qPCR SYBR Green Master Mix (Vazyme Biotech, Nanjing, China). Expression levels of PRRSV nsp9, IFN-β, IL-6, IL-8, and TNF-α were determined using the oligos listed in Table 1. The expression level of β-actin was also measured as an internal control. The relative RNA levels were calculated using the 2^−∆∆Ct^ method.

### 2.4. Western Blot Analysis

Cell lysates were harvested with IP lysis buffer (Biosharp, Hefei, China) supplemented with a protease inhibitor cocktail (Roche, Basel, Switzerland). Lysates were mixed with 5 × loading buffer and boiled at 95 °C for 5 min. Cell debris was removed by centrifugation at 12,000× *g* at 4 °C for 10 min. Cell lysates were mixed with 5 × loading buffer containing β-mercaptoethanol and heated at 95 °C for 5 min. After being separated by electrophoresis in 12% SDS-PAGE gel, all proteins in the gel were blocked onto a nitrocellulose membrane (Sangon Biotech, Shanghai, China). The membrane was blocked with 5% skim milk in 1 × PBS for 1 h at room temperature. The membranes were incubated with primary antibodies at 4 °C overnight. After extensive washes with 1 × PBS supplemented with 0.05% Tween 20, the membranes were incubated with HRP-conjugated secondary antibodies (anti-rabbit IgG or anti-mouse IgG) for 1 h at room temperature. The target protein bands were visualized with ECL substrate (Vazyme Biotech, Nanjing, China) using a Tanon 5200 Multi imaging system.

### 2.5. Immunofluorescence Assay (IFA)

An immunofluorescence assay was performed to visualize the expression of PRRSV N in virus-infected cells. MARC-145 cells infected with PRRSV were fixed with ice-cold methanol at 24 h post-infection (hpi). The cell monolayer was blocked with 1% bovine serum albumin in PBS for 1 h at room temperature and incubated with an appropriately diluted monoclonal antibody against PRRSV N at 37 °C for 1 h. Cells were washed with PBS five times and incubated with Alexa Fluor 488-conjugated goat anti-mouse secondary antibody at 37 °C for 1 h. The fluorescent signal was checked with an IX73 epifluorescence microscope (Olympus, Tokyo, Japan).

### 2.6. Gaussia Luciferase Assay

As described previously [23], a gaussia luciferase assay was performed to access viral load in the virus supernatants of TA-Gluc2. Briefly, mix 20 µL of culture supernatant with 50 μL coelenterazine h (20 μM) in a white plate and acquire photon counts for 10 s using a plate reader (Shanpu, Shanghai, China).

### 2.7. In Vitro Viricidal Assay

To evaluate the viricidal activity of allicin against PRRSV, the mixtures of TA-Gluc2 or NL1207 at an MOI of 1 and allicin at different concentrations (0, 10, 20, 40, 80, 100, 200 μg/mL) were incubated at 37 °C for 2 h and then used to inoculate MARC-145 cells. At 2 hpi, remove the inoculum and wash the cells three times with PBS. Cells were further maintained with MEM supplemented with 2% FBS. At 24 hpi, culture supernatants were harvested for viral RNA load quantification using qRT-PCR, and cells were fixed for the immunofluorescence assay.

### 2.8. Time Course Study of the Antiviral Effect of Allicin on PRRSV Infection

MARC-145 cells grown in 24-well plates were inoculated with TA-Gluc2 (MOI = 1) at 4 °C for 2 h. After being washed three times with PBS, cells were maintained in MEM supplemented with 2% FBS at 37 °C. Allicin at 100 μg/mL or 0.1% DMSO was added to cells at 0, 2, 4, and 6 h after the temperature shift to 37 °C, respectively. At 24 hpi, culture supernatants were collected to determine virus progeny by qRT-PCR targeting PRRSV ORF6.

### 2.9. Viral Attachment, Entry, Replication, Assembly, and Release Assays

For viral attachment assay, MARC-145 cells were cooled at 4 °C for 2 h and then inoculated with TA-Gluc2 at an MOI of 5 with allicin at 100 μg/mL or 0.1% DMSO at 4 °C for 2 h to facilitate viral attachment. After being washed five times with ice-cold PBS to remove unbound virions, cells were harvested for total cellular RNA extraction for the quantification of the attached virions by qRT-PCR.

For viral entry assay, the monolayer of MARC-145 cells in a 6-well plate was cooled at 4 °C for 2 h and then inoculated with TA-EGFP at an MOI of 10 at 4 °C. At 2 h later, cells were washed three times with PBS and maintained with MEM containing 100 μg/mL allicin or 0.1% DMSO at 37 °C. At 7 or 10 hpi, cells were harvested for Western blot analysis. The expression level of PRRSV nsp2 indicated by green fluorescence and Western blot detection can be used to evaluate viral entry.

For viral replication assay, BHK-21 cells in 24-well plates were transfected with the infectious cDNA clone pCMV-TA-Gluc2 at 1 µg/well using the lipofectamine 3000 transfection reagent according to the manufacturer’s instructions (ThermoFisher Scientific, Waltham, MA, USA). At 24 hpt, the culture supernatants were harvested for viral RNA extraction. The contamination of plasmid DNA in viral RNA was removed using a DNA-free^TM^ kit (ThermoFisher Scientific, Waltham, MA, USA). A qRT-PCR targeting PRRSV ORF6 was performed to quantify the progeny of the virus.

For viral assembly assay, MARC-145 cells were inoculated with TA-Gluc2 at an MOI of 1 with 100 μg/mL allicin or 0.1% DMSO at 37 ℃. At 2 h later, cells were washed three times with PBS to remove unbound virus particles and maintained in MEM supplemented with 100 μg/mL allicin or 0.1% DMSO. At 24 hpi, culture supernatants were collected and cells were harvested with FastPure Cell/Tissue Total RNA Isolation Kit V2 buffer RL (Vazyme Biotech, Nanjing, China) for the extraction of total cellular RNA or freeze-and-thaw to release intracellular virus particles. Cell debris was removed by centrifugation at 12,000× *g* for 15 min at 4 °C. Intracellular and extracellular infectious virus particles were determined by TCID50 assay using freeze-and-thaw cell supernatants and culture supernatant, respectively. Viral RNAs extracted from culture supernatant and total cellular RNAs were used to quantify the viral genome using qRT-PCR targeting the PRRSV nsp9 coding region. The relative packaging efficiency of the viral genome was indicated by infectious viral titers divided by the total viral genome, and the relative package efficiency without allicin treatment was set as 1.

For viral release assay, MARC-145 cells were inoculated with TA-Gluc2 at an MOI of 1 at 37 °C to achieve normal virus replication. At 8 hpi, cells were washed three times with PBS and maintained in MEM supplemented with 100 μg/mL allicin or 0.1% DMSO. At 2 or 4 h later, culture supernatants were collected and cells were freeze-and-thawed to release intracellular virus particles. Cell debris was removed by centrifugation at 12,000× *g* for 15 min at 4 °C. Intracellular and extracellular infectious virus particles were determined by the TCID_50_ assay using freeze-and-thaw cell supernatants and culture supernatants, respectively. The percentage of viruses released was indicated by the ratio between the extracellular virus titer and the total virus titer.

### 2.10. Transcriptome Analysis

MARC-145 cells infected with TA-Gluc2 at an MOI of 1 were treated with 100 μg/mL allicin or 0.1% DMSO, and MARC-145 cells without infection were used as a negative control. Three biological replicates were included for each treatment. At 24 hpi, cell lysates were harvested and total RNAs were extracted with TRIzol reagent (Vazyme Biotech, Nanjing, China). mRNA samples purified from total RNA were used for RNA sequencing by Seqhealth Technology Co., Ltd. (Wuhan, China). Stranded RNA sequencing libraries prepared with the KCTM Stranded mRNA Library Prep Kit for Illumina^®^ (Catalogue NO. DR08402, Wuhan Seqhealth Co., Ltd., Wuhan, China) were sequenced on the DNBSEQ-T7 sequencer (MGI Tech Co., Ltd., Shenzhen, China) with the PE150 model. Raw sequencing data were first filtered by Trimmomatic (version 0.36), low-quality reads were discarded, and the reads contaminated with adaptor sequences were trimmed. Clean data were mapped to the reference genome of Chlorocebus sabaeus from https://asia.ensembl.org/Chlorocebus_sabaeus/Info/Index (accessed on 20 December 2022) using STRA software (version 2.5.3a) with default parameters. The reads assigned to the exon regions of each gene were counted using featureCounts (Subread-1.5.1; Bioconductor) and then the RPKMs were calculated. Genes differentially expressed between groups were identified using the edgeR package (version 3.12.1). Differentially expressed genes (DEG) were determined using thresholds of |log2 (fold change)| > 1 and *p*-value < 0.05. The Kyoto encyclopedia of genes and genomes (KEGG) enrichment analysis for differentially expressed genes was implemented using DAVID Bioinformatics Resources [24] with a *p*-value < 0.05 to judge statistically significant enrichment. Common and unique DEGs between different treatments were identified by a Venn diagram (https://bioinfogp.cnb.csic.es/tools/venny/index.html (accessed on 20 March 2023)). The expression profiles of DEGs in the TNF signaling pathway and MAPK signaling pathway were visualized by heatmaps generated with Clustvis [25].

### 2.11. Statistical Analysis

Statistical analyses were performed with GraphPad Prism software 8.0. Data are presented as mean values ± SD from at least three replicates. The mean values were compared using two-tailed Student’s *t*-tests or ANOVA with Tukey’s post hoc test and considered significantly different when *p*-value < 0.05.

## 3. Results

### 3.1. Allicin Inhibits the Infection of HP-PRRSV and NADC30-like PRRSV Strains in MARC-145 and PAM

**Figure 1:**
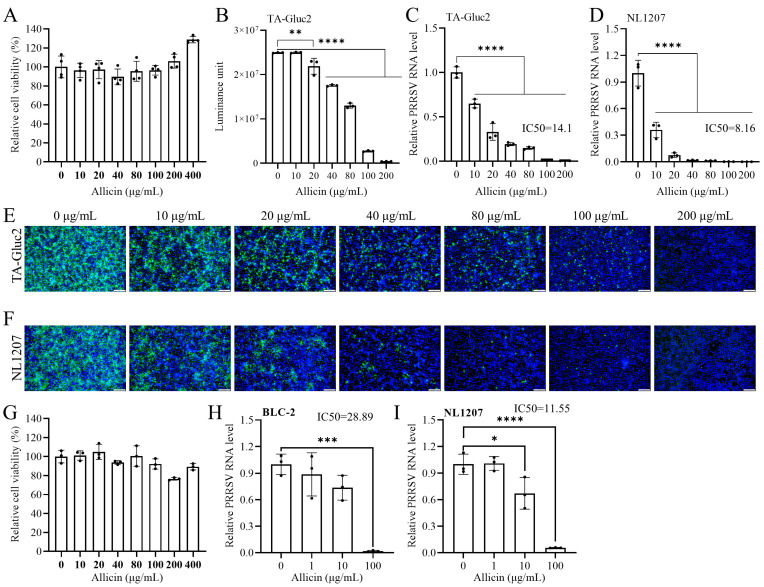
The anti-PRRSV activity of allicin in MARC-145 cells and PAM. (**A**) The cytotoxicity of allicin on MARC-145 cells. MARC-145 cells were treated with allicin at various doses ranging from 0 μg/mL to 400 μg/mL for 24 h. The cytotoxicity of allicin was determined using a CCK-8 assay. (**B**) Allicin inhibited TA-Gluc2 infection in a dose-dependent manner. MARC-145 cells were infected with the TA-Gluc2 reporter virus at a dose of 1 MOI. At 24 hpi, culture supernatants were harvested for the gaussia luciferase assay. (**C**,**D**) MARC-145 cells were infected with TA-Gluc2 or NL1207 at a dose of 1 MOI in the presence of allicin at different concentrations (0, 10, 20, 40, 80, 100, 200, and 400 μg/mL) for 24 h. Viral loads in culture supernatants were determined by qRT-PCR targeting PRRSV ORF6. PRRSV infection was also monitored by IFA detection of PRRSV N in virus-infected cells, and cell nuclei were counterstained with DPAI (**E**,**F**). (**G**) The cytotoxicity of Allicin on PAMs. PAMs were incubated with allicin at various doses ranging from 0 to 100 μg/mL for 24 h. A CCK-8 assay was performed to examine the effect of allicin on the viability of PAM. (**H**,**I**) PAMs were infected with BLC-2 or NL1207 at a dose of 1 MOI in the presence of allicin at different concentrations (0, 1, 10, and 100 μg/mL) for 24 h. Viral loads in culture supernatants were determined by qRT-PCR targeting PRRSV ORF6. *, *p*-value < 0.05; **, *p*-value < 0.01; ***, *p*-value < 0.001; ****, *p*-value < 0.0001.

The cytotoxicity of allicin to MARC-145 cells was evaluated using the CCK-8 assay. No statistically significant cytotoxicity was observed with allicin treatment at tested concentrations of 10~400 μg/mL (Figure 1A). Interestingly, allicin at 200~400 μg/mL slightly increased the viability of MARC-145 cells compared to the DMSO-treated control. We initially evaluated the inhibitory effect of allicin on PRRSV infection in MARC-145 cells using a reporter virus TA-Gluc2 (HP-PRRSV) previously described [8]. Since Gluc encoded by the reporter virus in virus-infected cells is a naturally secreted protein, we evaluated the antiviral effect of allicin by a gaussia luciferase assay using culture supernatants. As shown in Figure 1B, the expression level of Gluc was down-regulated dose-dependent by allicin treatment. In line with the expression of Gluc, viral RNA loads in the culture supernatants determined by qRT-PCR were reduced in a dose-dependent manner by allicin treatment with an IC_50_ of 14.1 μg/mL (Figure 1C). The expression of PRRSV N protein in virus-infected cells detected by IFA was also suppressed similarly by allicin treatment (Figure 1E). To rule out that the anti-PRRSV effect of allicin is strain-specific, we further evaluated the sensitivity of a NADC30-like PRRSV strain NL1207 to allicin treatment. A similar inhibitory effect of allicin on the NL1207 strain was indicated by the dose-dependent down-regulated viral RNA loads in the culture supernatants, and the IC50 was 8.16 μg/mL (Figure 1D). Consistently, the expression of PRRSV N was also suppressed by allicin treatment (Figure 1F).

PRRSV primarily infects porcine alveolar macrophage in vivo. The antiviral effect of allicin on PRRSV infection was also confirmed using PAM isolated from 3-week piglets. No cytotoxicity effect was observed when the concentration of allicin was not greater than 100 μg/mL (Figure 1G). The antiviral effect of allicin on HP-PRRSV strain BLC-2 and the NL1207 strain was evaluated based on viral RNA loads in culture supernatants. According to data generated with PRRSV infection with MARC-145 cells, the infection of both strains was suppressed in a dose-dependent manner (Figure 1H,I). The IC_50_ values against BLC-2 and NL1207 were 28.89 μg/mL and 11.55 μg/mL, respectively.

### 3.2. Allicin Does Not Have a Significant Virucidal Effect on PRRSV

**Figure 2:**
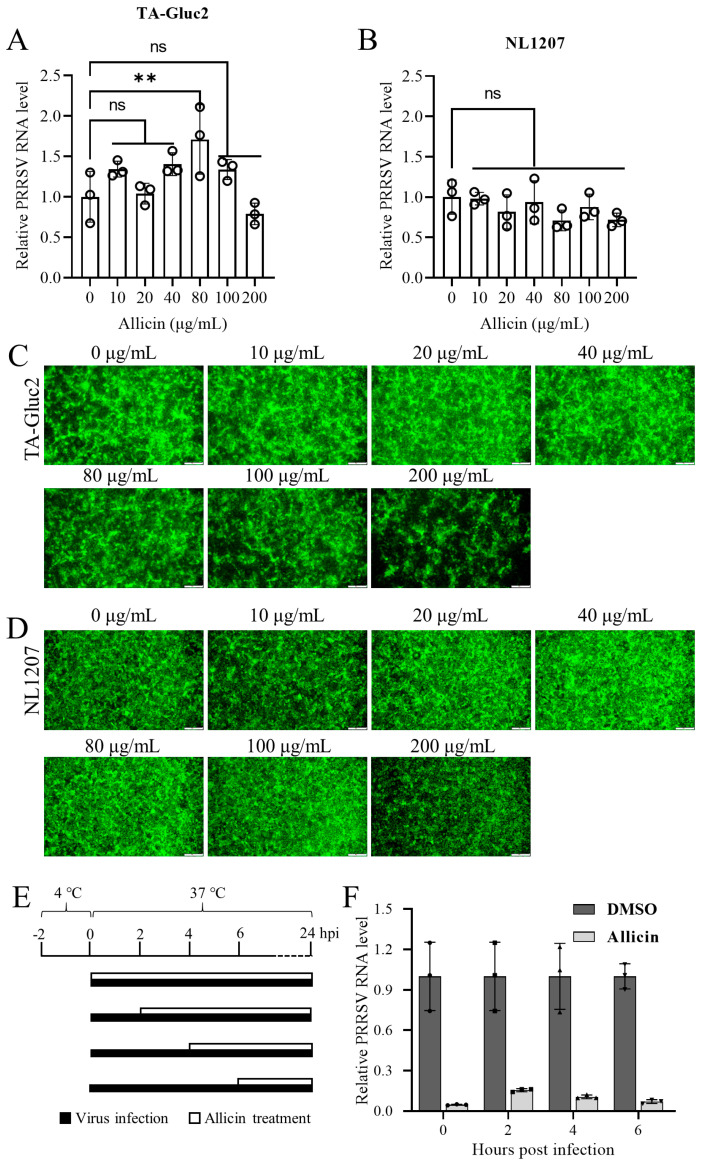
Allicin exhibits inhibitory effects on multiple stages of the PRRSV life cycle. A dose of 1 MOI TA-Gluc2 (**A**) or NL1207 (**B**) pre-incubated with allicin at different concentrations (0, 10, 20, 40, 80, 100, 200 μg/mL) at 37 °C for 2  h was used to infect MARC-145 cells. At 24 hpi, culture supernatants were harvested for quantification of virus progeny by qRT-PCR targeting PRRSV ORF6. PRRSV infection was also monitored by IFA detection of PRRSV N in cells infected with TA-Gluc2 (**C**) or NL1207 (**D**). (**E**) A schematic diagram of the time course study of allicin’s inhibitory effect on PRRSV infection. (**F**) A time-of-drug addition assay. MARC-145 cells were infected with PRRSV TA-Gluc2 at an MOI of 1, and the culture supernatants were replaced with fresh medium supplemented with 100  μg/mL allicin or 0.1% DMSO at 2, 4, and 6 hpi. At 24 hpi, culture supernatants were collected for the quantification of virus progeny by qRT-PCR targeting PRRSV ORF6. **, *p*-value < 0.01; ns, not statistically significant.

To understand whether allicin has a direct viricidal effect on PRRSV, we pretreated TA-Gluc2 and NL1207 with allicin at different concentrations at 37 °C for 2 h before infection. The infectivity of pretreated viruses to MARC-145 cells was evaluated by virus progeny in culture supernatant at 24 hpi. As shown in Figure 2A,B, the treatments of allicin at concentrations ranging from 10 to 200 μg/mL did not exhibit a significant inhibitory effect on virus progeny, suggesting that allicin does have a significant virucidal effect on PRRSV. Virus infection was also confirmed by the detection of PRRSV N expression in virus-infected cells (Figure 2C,D).

### 3.3. Allicin Exhibits Inhibitory Effects on Multiple Stages of the PRRSV Life Cycle

The PRRSV life cycle can be divided into five stages, including attachment, internalization, replication, assembly, and release. Each stage could be targeted by allicin to block PRRSV infection. To identify the specific stages of the PRRSV life cycle affected by allicin treatment, a study of the time course was carried out as depicted in Figure 2E. Allicin treatments were started at 0, 2, 4, or 6 hpi. At 24 hpi, virus progenies in culture supernatants were quantified by qRT-PCR. All allicin treatments exhibited strong antiviral effects on PRRSV infection indicated by the significantly reduced viral RNA loads (Figure 2F). These results suggested that allicin can suppress PRRSV infection at multiple stages of its life cycle.

### 3.4. Allicin Alleviates PRRSV Entry, but Not Attachment

**Figure 3:**
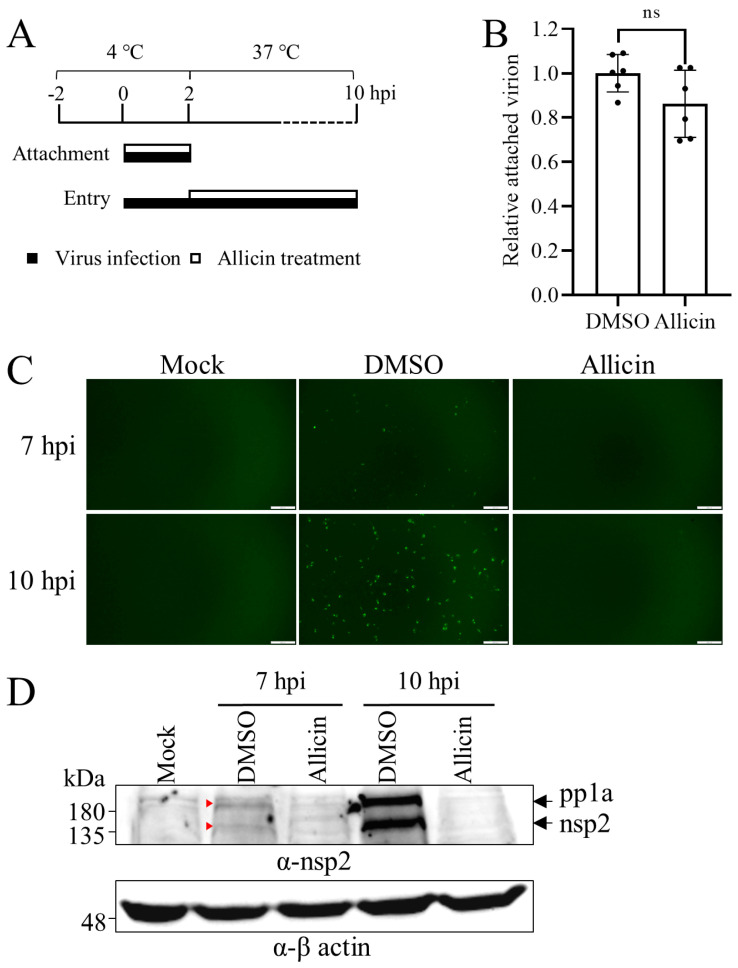
The effects of allicin on the attachment and entry steps of PRRSV infection. (**A**) Schematic diagram of attachment and entry experiments for PRRSV infection. For viral attachment assay, MARC-145 cells were cooled at 4 °C for 2 h and then inoculated with TA-Gluc2 at an MOI of 5 with allicin at 100 μg/mL or 0.1% DMSO at 4 °C for 2 h to facilitate viral attachment. After being washed five times with ice-cold PBS to remove unbound virions, cells were harvested for total cellular RNA extraction for the quantification of the attached virions by qRT-PCR targeting PRRSV ORF6 (**B**). For viral entry assay, the confluent monolayers of MARC-145 cells in a 6-well plate were cooled at 4 °C for 2 h and then inoculated with TA-EGFP at an MOI of 10 at 4 °C. At 2 h later, cells were washed three times with PBS and maintained with MEM containing 100  μg/mL allicin or 0.1% DMSO at 37 °C. At 7 or 10 hpi, cells were harvested for Western blot analysis. The expression level of PRRSV nsp2 indicated by green fluorescence (**C**) and Western blot detection (**D**) was used to evaluate viral entry, and the expression of pp1a and nsp2 at 7 hpi was highlighted with red triangles. ns, not statistically significant.

Initially, we determined the effect of allicin treatment on the attachment and entry steps of PRRSV infection as depicted in Figure 3A. MARC-145 cells were incubated with 5 MOI of TA-Gluc2 with or without allicin treatment at 100 μg/mL at 4 °C for 2 h and thoroughly washed with cold PBS to remove unbound viral particles. No significant difference between the allicin treatment and control was observed (Figure 3B), suggesting that allicin did not significantly affect PRRSV attachment.

Next, we evaluated the effect of allicin treatment on PRRSV entry. After viral attachment and internalization, the viral genomic RNA released into the cytoplasm directs the translation of PRRSV polyprotein 1a (pp1a), which will be further proteolytically processed into mature nonstructural proteins (nsp1α/β, nsp2, etc.). We employed a reporter virus TA-EGFP to evaluate PRRSV entry by monitoring the expression of nsp2 at the early stage of PRRSV infection. The EGFP gene inserted at the hypervariable region of nsp2 is expressed as a fusion protein (nsp2-EGFP) in virus-infected cells. The expression of nsp2-EGFP began at about 7 hpi, indicated by the green fluorescent signal in infected cells and was further enhanced at 10 hpi (Figure 3C). The green fluorescent signal was much stronger in cells without allicin treatment than that in cells treated with allicin. Western blot analysis of nsp2-EGFP expression was also performed with a polyclonal antibody against nsp2. Two proteins detected in cells without allicin treatment should be nsp2 and pp1a based on their sizes, which almost disappeared in cells treated with allicin (Figure 3D). These results suggested that allicin could inhibit viral entry. Since CD163 serves as the main receptor for PRRSV internalization, we also studied the effect of allicin on CD163 expression in PAMs. Unexpectedly, allicin treatment (10~40 μg/mL) for 12 h slightly increased the expression of CD163 in PAM (Figure S1).

### 3.5. Allicin Suppresses PRRSV Replication in BHK-21 Cells

**Figure 4:**
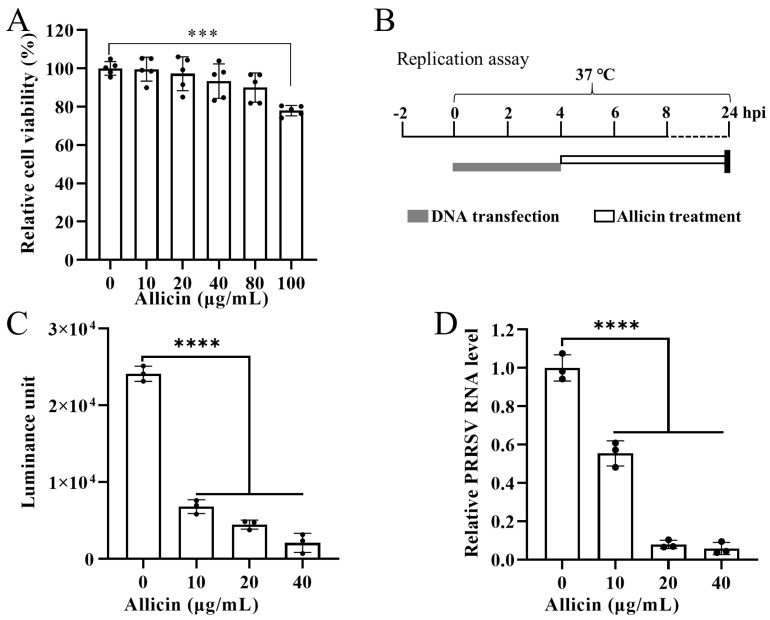
Allicin inhibits viral replication during PRRSV infection. (**A**) A schematic diagram of the replication assay. (**B**) The cytotoxicity effect of allicin on BHK-21 cells. BHK-21 cells were treated with allicin at various doses ranging from 0 μg/mL to 100 μg/mL for 24 h. Then, the cytotoxicity of allicin was determined using a CCK-8 assay. BHK-21 cells in 24-well plates were transfected with the infectious cDNA clone pCMV-TA-Gluc2 at 1 µg/well. At 4 hpt, cells were treated with allicin at different concentrations (0, 10, 20, or 40 μg/mL). At 24 hpt, culture supernatants were harvested for the gaussia luciferase assay and viral RNA extraction. The viral loads in the culture supernatants were determined by the expression levels of gaussia luciferase (**C**) and a qRT-PCR targeting PRRSV ORF6 (**D**). ***, *p*-value < 0.001; ****, *p*-value < 0.0001.

To exclude viral attachment and entry, the effect of allicin treatment on viral replication was investigated using DNA transfection of BHK-21 cells with a PRRSV cDNA clone pCMV-TA-Gluc2. Based on the CCK8 assay, allicin at 10~80 μg/mL exhibited no significant cytotoxicity to BHK-21 cells (Figure 4A). As depicted in Figure 4B, BHK-21 cells transfected with pCMV-TA-Gluc2 were treated with allicin at 10~40 μg/mL or mock treated for 20 h. Since Gluc expression is correlated with viral replication of TA-Gluc2 and Gluc is a naturally secreted protein [8], a gaussia luciferase assay was conducted to detect Gluc in culture supernatants. The expression of Gluc was significantly inhibited by allicin treatment in a dose-dependent manner (Figure 4C). Consistently, virus progenies in culture supernatants were also reduced by allicin treatment (Figure 4D).

### 3.6. Allicin Blocks the Packaging of the PRRSV Genome but Does Not Inhibit the Release of PRRSV Progeny

**Figure 5:**
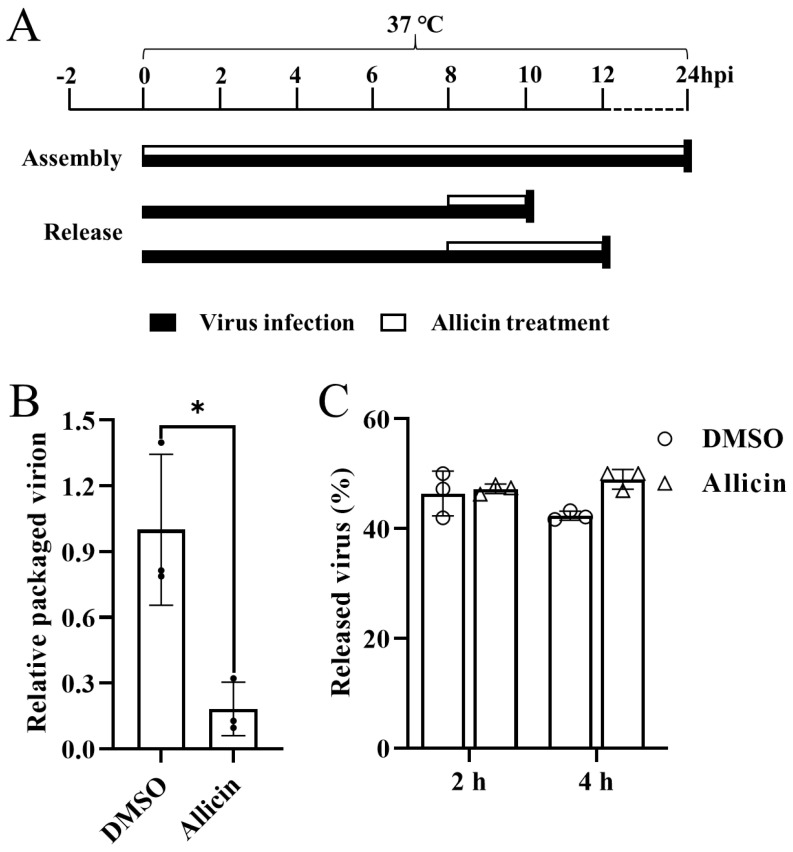
The effects of allicin on the PRRSV assembly and release. (**A**) A schematic diagram of the assembly and release assays. For viral assembly assay, MARC-145 cells were inoculated with TA-Gluc2 at an MOI of 1 with 100 μg/mL allicin or 0.1% DMSO at 37 °C. At 2 h later, cells were washed three times with PBS to remove unbound virus particles and maintained in MEM supplemented with 100 μg/mL allicin or 0.1% DMSO. Culture supernatants and cells were collected at 24 hpi. Intracellular and extracellular infectious virus particles were determined by TCID_50_ assay, and total viral genomes were quantified by qRT-PCR targeting the PRRSV nsp9 coding region. The relative packaging efficiency of the viral genome was indicated by infectious viral titers divided by the total viral genome, and the relative package efficiency without allicin treatment was set as 1 (**B**). For viral release assay, MARC-145 cells were inoculated with TA-Gluc2 at an MOI of 1 at 37 °C to achieve normal virus replication. At 8 hpi, cells were washed three times with PBS and maintained in MEM supplemented with 100 μg/mL allicin or 0.1% DMSO. At 2 or 4 h later, the intracellular and extracellular infectious virus particles were determined by the TCID_50_ assay using the freeze-and-thaw cell supernatants and culture supernatants, respectively. The percentage of viruses released was indicated by the ratio between the extracellular virus titer and the total virus titer (**C**). *, *p*-value < 0.05.

Subsequently, we further determined the effect of allicin on post-replication events of the PRRSV life cycle. The assembly and release assays were performed as illustrated in Figure 5A. The relative packaging efficiency of the viral genome was calculated by dividing the infectious viral titers by the total viral genome. As shown in Figure 5B, the ratio of the packaged genome was significantly reduced by allicin treatment, suggesting that allicin exhibits an inhibitory effect on PRRSV assembly. We also evaluated the release of mature virions using a viral release assay as depicted in Figure 5A. MARC-145 cells inoculated with TA-Gluc2 for 8 h to achieve normal virus replication were treated with MEM supplemented with 100 μg/mL allicin or 0.1% DMSO. At 2 or 4 h later, the intracellular and extracellular infectious virus particles were determined by the TCID_50_ assay. The percentage of viruses released was indicated by the ratio between the extracellular virus titer and the total virus titer. Similar percentages of released viruses were observed in cells with or without allicin treatment (Figure 5C). Therefore, allicin does not inhibit the release of PRRSV progeny.

### 3.7. Allicin Ameliorates PRRSV-Induced Inflammatory Responses

**Figure 6:**
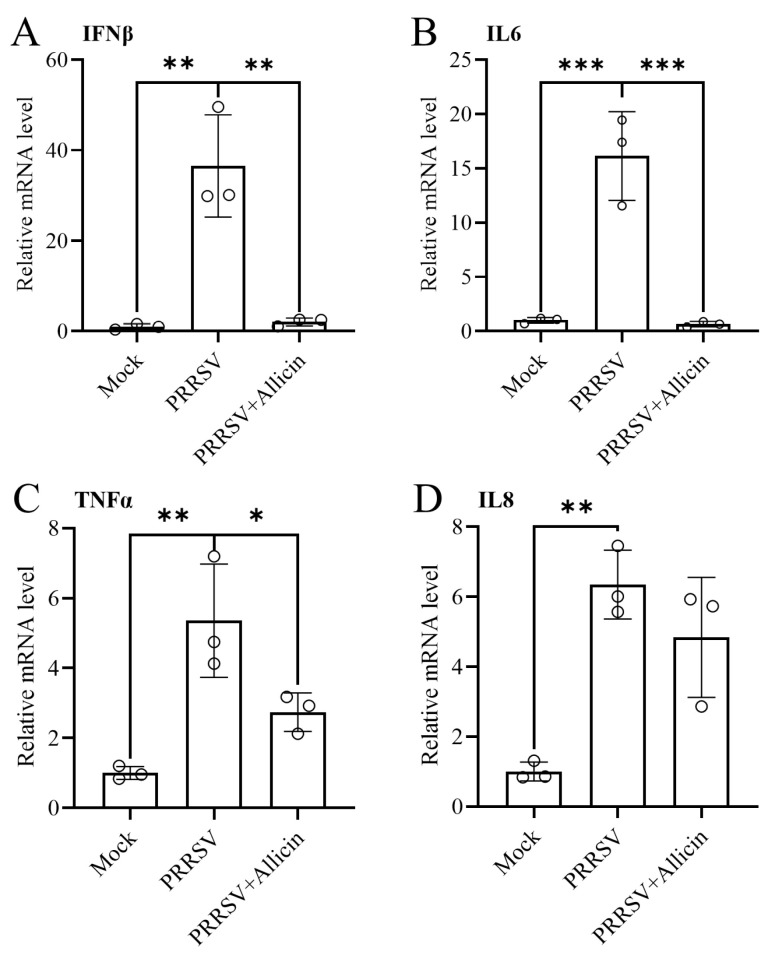
Allicin reduced PRRSV-induced pro-inflammatory cytokine responses. MARC-145 cells were mock infected or infected with TA-Gluc2 at an MOI of 1 in the presence of 100 μg/mL allicin or 0.1% DMSO. At 24 hpi, qRT-PCR was performed to assess the expression of four pro-inflammatory cytokines, including IFN-β (**A**), IL-6 (**B**), TNF-α (**C**), and IL-8 (**D**). The expression of β-actin was determined as an internal control. *, *p*-value < 0.05; **, *p*-value < 0.01; ***, *p*-value < 0.001.

PRRSV infection induces the release of pro-inflammatory cytokines and causes the interferon response, which influences host immune responses and pathology [26,27]. Since a “cytokine storm” caused by PRRSV infection contributes to disease pathogenesis and lung damage [2,28], anti-inflammation could be another important property of an anti-PRRSV drug candidate. Allicin has immunomodulatory properties and inhibits the release of pro-inflammatory cytokines, such as IL-6 and TNF-α [29,30]. To study the effect of allicin on the inflammatory responses caused by PRRSV infection, we evaluated the expression of pro-inflammatory cytokines at the mRNA level. MARC-145 cells with PRRSV infection were treated with 100 μg/mL allicin or mock treated for 24 h, and qRT-PCR was performed to assess the expression of IFN-β, IL-6, TNF-α, and IL-8. As shown in Figure 6, the up-regulated expression of pro-inflammatory cytokines elicited by PRRSV infection was alleviated by allicin treatment.

**Figure 7:**
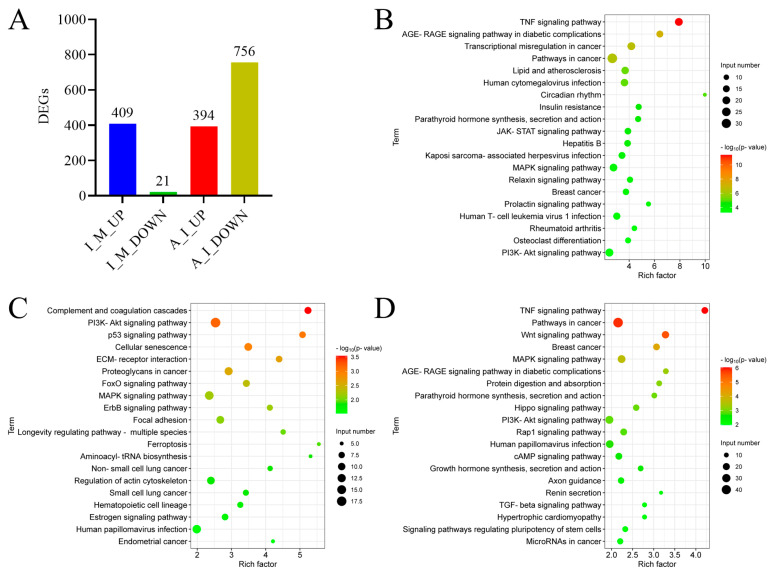
Bioinformatic analysis of the DEGs in PRRSV-infected MARC-145 cells. MARC-145 cells were mock infected or infected with TA-Gluc2 at an MOI of 1 in the presence of 100 μg/mL allicin or 0.1% DMSO. At 24 hpi, total cellular RNA was extracted for transcriptome analysis by RNA sequencing. (**A**) The up-regulated and down-regulated DEGs were identified between infection and mock (I_M_UP and I_M_DOWN) and between allicin/infection and infection (A_I_UP and A_I_DOWN). Top-enriched KEGG pathways were identified for the up-regulated DEG in PRRSV-infected cells (**B**), the up-regulated DEGs in cells with PRRSV infection and allicin treatment (**C**), and the down-regulated DEGs in cells with PRRSV infection and allicin treatment (**D**).

**Figure 8:**
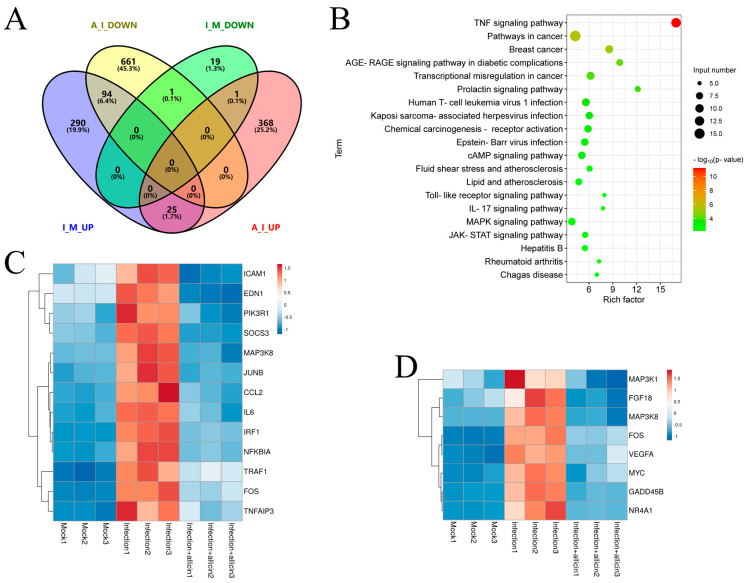
The DEGs up-regulated by PRRSV infection were restored by allicin treatment. (**A**) The common and unique DEGs were identified by a Venn diagram. (**B**) Top enriched KEGG pathways for DEGs that were up-regulated by PRRSV infection and restored by allicin treatment. The expression profiles of DEGs identified in MAPK signaling pathway (**C**) and TNF signaling pathway (**D**) were visualized by heatmaps plotted with Z scores indicated by a scale bar. The positive and negative Z scores indicate up-regulation and down-regulation, respectively.

To further explore the mechanism of allicin’s anti-inflammatory effect during PRRSV infection, we performed transcriptome analysis to identify DEG and the enriched pathways in virus-infected cells with or without allicin treatment. An amount of 409 up-regulated and 21 down-regulated DEGs were identified between PRRSV infection and mock, while 394 up-regulated and 756 down-regulated DEGs were identified between PRRSV infection and PRRSV infection/allicin treatment (Figure 7A and Figure S2). Based on the KEGG pathway enrichment analysis for DEG, the TNF signaling pathway and the MAPK signaling pathway, which are associated with inflammatory responses, were stimulated by PRRSV infection but restored by allicin treatment (Figure 7B,D). Furthermore, we identified 94 DEGs that were up-regulated by PRRSV infection but down-regulated by allicin treatment (Figure 8A). The enrichment analysis of the KEGG pathway for these DEGs confirmed that allicin treatment reduced the stimulation of the TNF signaling pathway and the MAPK signaling pathway (Figure 8B). As shown in Figure 8C,D, 13 DEGs and 8 DEGs enriched in the TNF signaling pathway and the MAPK signaling pathway were up-regulated by PRRSV infection and restored by allicin treatment. Among these DEGs, we found the serine/threonine kinases MAP3K1 and MAP3K8 that are involved in the activation of the ERK and JNK kinase pathways as well as the NF-κB pathway. Taken together, our results suggest that allicin may exert its anti-inflammatory effect during PRRSV infection by suppressing the TNF and MAPK signaling pathways.

## 4. Discussion

With the increasing public health issues caused by infectious diseases in recent years, the active ingredients in traditional medicinal plants are receiving increasing attention. Since herbs and their extracts have long been used for the treatment of various diseases, including viral infections, the active ingredients in their extracts are excellent precursors for new antiviral drugs. As one of the oldest herbs since ancient times, garlic is commonly used in the treatment of several common diseases such as colds, snake bites, indigestion, wound ulcers, hypertension, and respiratory and genitourinary tract infections [31]. The active ingredients in garlic also have a variety of biological activities, including anticancer [32], antioxidant [33], antiatherosclerotic [34], and antibacterial and antiviral activity [35]. As the most biologically active organosulfur compound in garlic, allicin exhibited antiviral activity against many animal viruses, such as the influenza virus and SARS-CoV-2. In this study, we investigated the antiviral effect of allicin on PRRSV infection. As expected, allicin significantly inhibited the infection of two PRRSV strains prevalent in China. The antiviral activity of allicin in PRRSV is achieved by blocking viral entry into host cells, as well as the replication and assembly of the viral genome. In addition, the pro-inflammatory responses induced by PRRSV infection were alleviated by allicin treatment, which was supported by the down-regulated expression of pro-inflammatory cytokines and restored inflammatory-associated pathways in cells with allicin treatment.

Targeting the attachment or entry of the virus to host cells would be a potential antiviral mechanism of allicin. GE and its OSCs exert their antiviral activity through interaction with the viral envelope or host cell surface charge molecule, which leads to the blockage or suppression of viral entry into host cells [15]. For instance, GE in gold nanoparticles interacts with viral surface proteins which leads to its virucidal activity against the measle virus by blocking viral entry into host cells [36]. Allicin was reported as one of the main OSCs that is responsible for the antiviral activity of GE [18,20]. Here, we also observed that the entry of PRRSV into MARC-145 cells was inhibited by allicin. However, allicin has no virucidal activity against PRRSV. Based on our results, allicin may interact with the host cell surface charge molecule to block viral entry but not with the viral envelope. Allicin prevents the entry of SARS-CoV-2 by targeting endocytosis-related proteins, such as KIFA/B/C kinesins, clathrin CLTCL1, and tubulin TUBAL3 [19]. Rab5 (a marker of early endosomes) and Rab7 (a marker of late endosomes) are required for the entry of many viruses into host cells through the clathrin-dependent pathway [37,38,39,40,41,42]. However, the expression of Rab5 and Rab7 was not affected in MARC-145 cells by allicin treatment [43]. Allicin reduces intracellular levels of reduced glutathione and modifies it by S-thioallylation [34]. Since viral entry and membrane fusion require the reduced state of the host cell cytoplasm, allicin can also regulate viral infection by affecting the host cell’s redox state and the abundance of specific proteins involved in membrane trafficking, motility, and tight junction [19]. In addition, given cellular cholesterol is required for PRRSV entry and the cholesterol-lowering effect of allicin [44,45], whether allicin reduces cellular cholesterol to suppress PRRSV infection should be explored in future studies.

Inhibition of viral replication and assembly is another potential antiviral mechanism of allicin [15]. Since the biological effects of allicin involve the S-thioallylation of proteins [46], allicin could target Cys-containing viral proteins. Several reporters suggested that allicin may induce dual S-thioallylation of the catalytic active sites (Cys-145 and solvent-exposed Cys-85/Cys-156 residues) of SARS-CoV-2 main protease, thus affecting the protease activity and inhibiting virus replication, although viral S-thioallylated peptides were not detected by proteomics and mass spectrometry [19,47,48]. Since PRRSV papain-like protease PLP2, main protease nsp4, and RNA-dependent RNA polymerase nsp9 are Cys-rich viral proteins and critical for viral replication, they could be targeted by allicin for S-thioallylation. To test this possibility, the effect of allicin on the protease activity of PRRSV nsp4 was tested. However, the protease activity of nsp4 was not affected by allicin treatment in a reporter assay [49]. We could do an in silico docking study to identify the potential S-thioallylation of other viral Cys proteins and test the inhibitory effects of allicin on them in future studies.

Immunomodulating properties of natural products and plant extracts usually play a crucial role in the alleviation of viral infection [50]. Allicin exerts an antiviral effect against the reticuloendotheliosis virus by restricting the inflammation and oxidative damage caused by the infection, especially by blocking the ERK/MAPK pathway [20]. Inhibiting the P38 and JNK pathways and the TLR4/NF-*κ*B signaling pathway was also considered as the anti-inflammatory mechanism of allicin [14,21]. In this study, the up-regulated expression of pro-inflammatory cytokines (IFN-β, IL-6, and TNF-α) by PRRSV infection was restored by allicin treatment. We also conducted transcriptome analysis to further characterize the anti-inflammation mechanism of allicin. In line with previous reporters, the down-regulated DEGs by allicin enriched the inflammatory-associated signaling pathways (TNF signaling pathway and MAPK signaling pathway) activated by PRRSV infection. Taken together, the anti-inflammation property of allicin also involves the antiviral mechanism against PRRSV.

## 5. Conclusions

In conclusion, our results demonstrate that allicin inhibits the viral entry, replication, and assembly of the viral life cycle of PRRSV in a dose-dependent way. In addition, allicin alleviates inflammation responses caused by PRRSV by suppressing the TNF and MAPK signaling pathways. This study supports that allicin is a promising drug candidate for controlling PRRSV and warrants further investigation in vivo.

## Figures and Tables

**Table 1:** A list of primers and probes for qRT-PCR.

Gene	Primer	Probe
PRRSV-ORF6	CGGCAARTGATAACCACGC	GTCGTCCGGCGTCCCGG
	TGCCACCCAACACGAGG	
PRRSV-nsp9	CGGCGGCTTAGTTGTTACTG	
	TCAACCTCACTGGCCACTTT	
IFN-β	CCTGAAGGCCAAGGAGTACA	CCACTCTGACTATGGTCCAGGCACA
	AGCAATTGTCCAGTCCCAGA	
IL-6	CAGCCCTGAGAAAGGAGACA	TGCCAGTGCCTCTTTGCTGCT
	CCAGGCAAGTCTCCTCATTG	
TNFα	TCCCCAGGAAGACAGCG	
	GCAGCAGACAGAAGAGCGT	
IL8	ACTCCAAACCTATCCACCCC	
	CCACAACCCTAGACACCCAT	
β-actin	CCCTGGAGAAGAGCTACGAG	CAGGAAGGAAGGTTGGAAGAG
	CGGTTCCGCTGCCCTGAGGC	

## Data Availability

The datasets generated for this study are available on request to the corresponding author.

## Supplementary Materials

The following supporting information can be downloaded at: https://www.mdpi.com/article/10.3390/v15051050/s1, Figure S1: Allicin exhibits no inhibitory effect on CD163 expression in PAMs; Figure S2: Volcano plot analysis of the differentially expressed genes induced by PRRSV infection or allicin treatment.

## References

[1] Thanawongnuwech R., Thacker B., Halbur P., Thacker E.L. (2004). Increased production of proinflammatory cytokines following infection with porcine reproductive and respiratory syndrome virus and Mycoplasma hyopneumoniae. Clin. Diagn. Lab. Immunol..

[2] Li J., Wang S., Li C., Wang C., Liu Y., Wang G., He X., Hu L., Liu Y., Cui M. (2017). Secondary Haemophilus parasuis infection enhances highly pathogenic porcine reproductive and respiratory syndrome virus (HP-PRRSV) infection-mediated inflammatory responses. Vet. Microbiol..

[3] Wensvoort G., Terpstra C., Pol J.M., ter Laak E.A., Bloemraad M., de Kluyver E.P., Kragten C., van Buiten L., den Besten A., Wagenaar F. (1991). Mystery swine disease in The Netherlands: The isolation of Lelystad virus. Vet. Q..

[4] Hanada K., Suzuki Y., Nakane T., Hirose O., Gojobori T. (2005). The origin and evolution of porcine reproductive and respiratory syndrome viruses. Mol. Biol. Evol..

[5] Murtaugh M.P., Stadejek T., Abrahante J.E., Lam T.T., Leung F.C. (2010). The ever-expanding diversity of porcine reproductive and respiratory syndrome virus. Virus Res..

[6] Cui X., Xia D., Huang X., Sun Y., Shi M., Zhang J., Li G., Yang Y., Wang H., Cai X. (2022). Analysis of Recombinant Characteristics Based on 949 PRRSV-2 Genomic Sequences Obtained from 1991 to 2021 Shows That Viral Multiplication Ability Contributes to Dominant Recombination. Microbiol. Spectr..

[7] Tian K., Yu X., Zhao T., Feng Y., Cao Z., Wang C., Hu Y., Chen X., Hu D., Tian X. (2007). Emergence of fatal PRRSV variants: Unparalleled outbreaks of atypical PRRS in China and molecular dissection of the unique hallmark. PLoS ONE.

[8] Li Y., Ren C., Li C., Xiao Y., Zhou Y. (2022). A Recombinant Porcine Reproductive and Respiratory Syndrome Virus Stably Expressing a Gaussia Luciferase for Antiviral Drug Screening Assay and Luciferase-Based Neutralization Assay. Front. Microbiol..

[9] Xu H., Li C., Li W., Zhao J., Gong B., Sun Q., Tang Y.D., Xiang L., Leng C., Peng J. (2022). Novel characteristics of Chinese NADC34-like PRRSV during 2020–2021. Transbound. Emerg. Dis..

[10] Chen X.X., Zhou X., Guo T., Qiao S., Guo Z., Li R., Jin Q., Hu X., Xing G., Deng R. (2021). Efficacy of a live attenuated highly pathogenic PRRSV vaccine against a NADC30-like strain challenge: Implications for ADE of PRRSV. BMC Vet. Res..

[11] Chai W., Liu Z., Sun Z., Su L., Zhang C., Huang L. (2020). Efficacy of two porcine reproductive and respiratory syndrome (PRRS) modified-live virus (MLV) vaccines against heterologous NADC30-like PRRS virus challenge. Vet. Microbiol..

[12] Nakamoto M., Kunimura K., Suzuki J.I., Kodera Y. (2020). Antimicrobial properties of hydrophobic compounds in garlic: Allicin, vinyldithiin, ajoene and diallyl polysulfides. Exp. Ther. Med..

[13] Catanzaro E., Canistro D., Pellicioni V., Vivarelli F., Fimognari C. (2022). Anticancer potential of allicin: A review. Pharmacol. Res..

[14] Li C., Lun W., Zhao X., Lei S., Guo Y., Ma J., Zhi F. (2015). Allicin alleviates inflammation of trinitrobenzenesulfonic acid-induced rats and suppresses P38 and JNK pathways in Caco-2 cells. Mediat. Inflamm..

[15] Rouf R., Uddin S.J., Sarker D.K., Islam M.T., Ali E.S., Shilpi J.A., Nahar L., Tiralongo E., Sarker S.D. (2020). Antiviral potential of garlic (Allium sativum) and its organosulfur compounds: A systematic update of pre-clinical and clinical data. Trends Food Sci. Technol..

[16] Borlinghaus J., Albrecht F., Gruhlke M.C., Nwachukwu I.D., Slusarenko A.J. (2014). Allicin: Chemistry and biological properties. Molecules.

[17] Lawson L.D., Hunsaker S.M. (2018). Allicin Bioavailability and Bioequivalence from Garlic Supplements and Garlic Foods. Nutrients.

[18] Weber N.D., Andersen D.O., North J.A., Murray B.K., Lawson L.D., Hughes B.G. (1992). In vitro virucidal effects of *Allium sativum* (garlic) extract and compounds. Planta Med..

[19] Mosbauer K., Fritsch V.N., Adrian L., Bernhardt J., Gruhlke M.C.H., Slusarenko A.J., Niemeyer D., Antelmann H. (2021). The Effect of Allicin on the Proteome of SARS-CoV-2 Infected Calu-3 Cells. Front. Microbiol..

[20] Wang L., Jiao H., Zhao J., Wang X., Sun S., Lin H. (2017). Allicin Alleviates Reticuloendotheliosis Virus-Induced Immunosuppression via ERK/Mitogen-Activated Protein Kinase Pathway in Specific Pathogen-Free Chickens. Front. Immunol..

[21] Che H.Y., Zhou C.H., Lyu C.C., Meng Y., He Y.T., Wang H.Q., Wu H.Y., Zhang J.B., Yuan B. (2023). Allicin Alleviated LPS-Induced Mastitis via the TLR4/NF-kappaB Signaling Pathway in Bovine Mammary Epithelial Cells. Int. J. Mol. Sci..

[22] Liu Y., Che T.M., Song M., Lee J.J., Almeida J.A., Bravo D., Van Alstine W.G., Pettigrew J.E. (2013). Dietary plant extracts improve immune responses and growth efficiency of pigs experimentally infected with porcine reproductive and respiratory syndrome virus. J. Anim. Sci..

[23] Tannous B.A. (2009). Gaussia luciferase reporter assay for monitoring biological processes in culture and in vivo. Nat. Protoc..

[24] Sherman B.T., Hao M., Qiu J., Jiao X., Baseler M.W., Lane H.C., Imamichi T., Chang W. (2022). DAVID: A web server for functional enrichment analysis and functional annotation of gene lists (2021 update). Nucleic Acids Res..

[25] Metsalu T., Vilo J. (2015). ClustVis: A web tool for visualizing clustering of multivariate data using Principal Component Analysis and heatmap. Nucleic Acids Res..

[26] Zhang M., Du T., Long F., Yang X., Sun Y., Duan M., Zhang G., Liu Y., Zhou E.M., Chen W. (2018). Platycodin D Suppresses Type 2 Porcine Reproductive and Respiratory Syndrome Virus in Primary and Established Cell Lines. Viruses.

[27] Zhang M., Wu Q., Chen Y., Duan M., Tian G., Deng X., Sun Y., Zhou T., Zhang G., Chen W. (2018). Inhibition of proanthocyanidin A2 on porcine reproductive and respiratory syndrome virus replication in vitro. PLoS ONE.

[28] Wang G., Song T., Yu Y., Liu Y., Shi W., Wang S., Rong F., Dong J., Liu H., Cai X. (2011). Immune responses in piglets infected with highly pathogenic porcine reproductive and respiratory syndrome virus. Vet. Immunol. Immunopathol..

[29] Shin J.H., Ryu J.H., Kang M.J., Hwang C.R., Han J., Kang D. (2013). Short-term heating reduces the anti-inflammatory effects of fresh raw garlic extracts on the LPS-induced production of NO and pro-inflammatory cytokines by downregulating allicin activity in RAW 264.7 macrophages. Food Chem. Toxicol..

[30] Gu X., Wu H., Fu P. (2013). Allicin attenuates inflammation and suppresses HLA-B27 protein expression in ankylosing spondylitis mice. Biomed. Res. Int..

[31] Ayaz E., Alpsoy H.C. (2007). Garlic (*Allium sativum*) and traditional medicine. Turk. Parazitolojii Derg..

[32] Siegers C.P., Steffen B., Röbke A., Pentz R. (1999). The effects of garlic preparations against human tumor cell proliferation. Phytomedicine Int. J. Phytother. Phytopharm..

[33] Rahman K., Lowe G.M. (2006). Garlic and cardiovascular disease: A critical review. J. Nutr..

[34] El-Saber Batiha G., Magdy Beshbishy A., Wasef G.L., Elewa Y.H.A., Al-Sagan A.A.-S., Abd El-Hack M.E., Taha A.E., Abd-Elhakim Y.M., Prasad Devkota H. (2020). Chemical Constituents and Pharmacological Activities of Garlic (*Allium sativum* L.): A Review. Nutrients.

[35] Block E. (1985). The chemistry of garlic and onions. Sci. Am..

[36] Melendez-Villanueva M.A., Moran-Santibanez K., Martinez-Sanmiguel J.J., Rangel-Lopez R., Garza-Navarro M.A., Rodriguez-Padilla C., Zarate-Trivino D.G., Trejo-Avila L.M. (2019). Virucidal Activity of Gold Nanoparticles Synthesized by Green Chemistry Using Garlic Extract. Viruses.

[37] Wei X., She G., Wu T., Xue C., Cao Y. (2020). PEDV enters cells through clathrin-, caveolae-, and lipid raft-mediated endocytosis and traffics via the endo-/lysosome pathway. Vet. Res..

[38] Benarroch Y., Juttukonda L., Sabharwal V., Boateng J., Khan A.R., Yarrington C., Wachman E.M., Taglauer E. (2021). Differential Expression of Rab5 and Rab7 Small GTPase Proteins in Placental Tissues from Pregnancies Affected by Maternal Coronavirus Disease 2019. Clin. Ther..

[39] Zhang Y.N., Liu Y.Y., Xiao F.C., Liu C.C., Liang X.D., Chen J., Zhou J., Baloch A.S., Kan L., Zhou B. (2018). Rab5, Rab7, and Rab11 Are Required for Caveola-Dependent Endocytosis of Classical Swine Fever Virus in Porcine Alveolar Macrophages. J. Virol..

[40] Shi B.J., Liu C.C., Zhou J., Wang S.Q., Gao Z.C., Zhang X.M., Zhou B., Chen P.Y. (2016). Entry of Classical Swine Fever Virus into PK-15 Cells via a pH-, Dynamin-, and Cholesterol-Dependent, Clathrin-Mediated Endocytic Pathway That Requires Rab5 and Rab7. J. Virol..

[41] Zhao R., Shi Q., Han Z., Fan Z., Ai H., Chen L., Li L., Liu T., Sun J., Liu S. (2021). Newcastle Disease Virus Entry into Chicken Macrophages via a pH-Dependent, Dynamin and Caveola-Mediated Endocytic Pathway That Requires Rab5. J. Virol..

[42] Yang Q.Y., Yang Y.L., Tang Y.X., Qin P., Wang G., Xie J.Y., Chen S.X., Ding C., Huang Y.W., Zhu S.J. (2022). Bile acids promote the caveolae-associated entry of swine acute diarrhea syndrome coronavirus in porcine intestinal enteroids. PLoS Pathog..

[43] Hu J. (2023). The inhibitory effect of allicin on the expression of Rab5 and Rab7. College of Veterinary Medicine, Yangzhou University, Yangzhou, China.

[44] Lu Y., He Z., Shen X., Xu X., Fan J., Wu S., Zhang D. (2012). Cholesterol-lowering effect of allicin on hypercholesterolemic ICR mice. Oxid. Med. Cell Longev..

[45] Jeon J.H., Lee C. (2017). Cellular cholesterol is required for porcine nidovirus infection. Arch. Virol..

[46] Gruhlke M.C.H., Antelmann H., Bernhardt J., Kloubert V., Rink L., Slusarenko A.J. (2019). The human allicin-proteome: S-thioallylation of proteins by the garlic defence substance allicin and its biological effects. Free Radic. Biol. Med..

[47] Bastikar V.A., Bastikar A.V., Chhajed S.S. (2020). Understanding the Role of Natural Medicinal Compounds Such as Curcumin and Allicin against SARS-CoV-2 Proteins as Potential Treatment against COVID-19: An in silico Approach. J. Proteom. Bioinform..

[48] Shekh S., Reddy K.K.A., Gowd K.H. (2020). In silico allicin induced S -thioallylation of SARS-CoV-2 main protease. J. Sulfur Chem..

[49] Hu J. (2023). The inhibitory effect of allicin on protease activity of PRRSV nsp4. College of Veterinary Medicine, Yangzhou University, Yangzhou, China.

[50] Ganjhu R.K., Mudgal P.P., Maity H., Dowarha D., Devadiga S., Nag S., Arunkumar G. (2015). Herbal plants and plant preparations as remedial approach for viral diseases. Virusdisease.

