# Supplementary material for: Allicin Inhibits Porcine Reproductive and Respiratory Syndrome Virus Infection In Vitro and Alleviates Inflammatory Responses
Source: Viruses. 2023 Apr 25;15(5):1050. doi: 10.3390/v15051050 (PMC10220932; doi:10.3390/v15051050)
Supplement: Supplementary file 1 [file viruses-15-01050-s001.zip › viruses-2345328-supplementary.pdf]

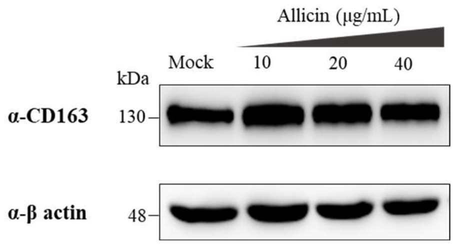

Figure S1. Allicin exhibits no inhibitory effect on CD163 expression in PAMs. PAMs were treated with allicin at concentrations of 10, 20, and 40 μg/mL or mock treated for 12 h. The expression of CD163 was evaluated by Western analysis, and the expression of β-actin was monitored as an internal control.

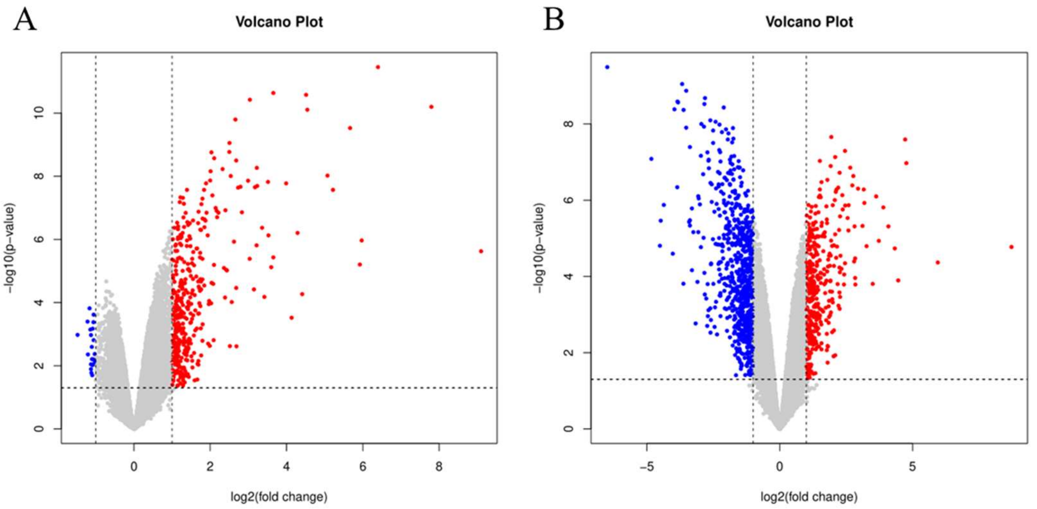

Figure S2. Volcano plot analysis of the differentially expressed genes induced by PRRSV infection or allicin treatment. Differentially expressed genes (DEG) were determined using thresholds of | log2 (fold change) | > 1 and p-value <0.05. (**A**) DEG between PRRSV infection and mock infection; (**B**) DEG between PRRSV infection/allicin treatment and PRRSV infection.
